# Implementation of concurrent electrolytic generation of two homogeneous mediators under widened potential conditions to facilitate removal of air-pollutants

**DOI:** 10.1038/s41598-017-00058-2

**Published:** 2017-02-13

**Authors:** Muthuraman Govindan, Alan M. Bond, Il-Shik Moon

**Affiliations:** 1 Department of Chemical Engineering, Sunchon National University, 255-Jungang ro, Suncheon-si, Jeollanam-do 57922 South Korea; 2 School of Chemistry, Monash University, Clayton, Victoria 3800 Australia

## Abstract

Electro-scrubbing is being developed as a futuristic technology for the removal of air-pollutants. To date, only one homogeneous mediator for the removal of air pollutants has been generated in each experiment using a divided electrolytic flow cell in an acidic medium. This paper reports the concurrent generation of two homogenous mediators, one at the anodic half-cell containing an acidic solution and the other at the cathodic half-cell containing a basic solution. The concept was inspired by the change in pH that occurs during water electrolysis in a divided cell. A 10 M KOH electrolyte medium assisted in the electrochemical generation of low valent 14% Co^1+^ ([Co^I^(CN)_5_]^4−^) mediator formed from reduction of [Co^II^(CN)_5_]^3−^ which was accompanied by a change in the solution 'oxidation reduction potential' (ORP) of −1.05 V Simultaneously, 41% of Co^3+^ was generated from oxidation of Co^II^SO_4_ in the anodic half-cell. No change in the solution ORP was observed at the cathodic half-cell when both half-cells contain 5 M H_2_SO_4_, and Co^3+^ was formed in the anodic half-cell. An electro-scrubbing approach based on the above principles was developed and tested on gaseous-pollutants, CH_3_CHO and CCl_4_, by Co^3+^ and Co^1+^, respectively, with 90 and 96% removal achieved, respectively.

## Introduction

The increasing concentration of atmospheric air-pollutants has prompted intensive research into the development of new environmentally friendly and affordable degradation methods. The generation of homogeneous mediators in aqueous media plays a key role in the mediated electrocatalytic oxidation (MEO) of air-pollutant degradation^1, 2^. Over the last decade, electrochemically oxidized metal ions, such as Ce^4+^, Ag^2+^ and Co^3+^, have been used as homogeneous mediators for pollutant degradation because of their very positive oxidation potentials, 1.4 V, 1.9 V and 1.8 V, respectively^3–5^, to generate what is considered as a futuristic technology by the United Nations Environmental Protection Agency (UN-EPA)^6^. In practice, these electrogenerated free metal ions, particularly Ag^2+^, are stable and soluble only under acid pH conditions (<1)^7^. Ce^4+^ and Co^3+^ have been generated in both divided and undivided cells, but Ag^2+^ can only generated in a divided cell because of its deposition as Ag metal on the cathode^8^.

In principle, reduced metal complexes could be useful for air-pollutant degradation. However, the H_2_ evolution potential (0.0 V) is well below the standard redox potential of the low oxidation states or metals of Ce^0^ (−2.33 V) and Co^0^ (−0.28 V)^9^, which restricts reduction of the simple metal ions to lower oxidation states under acidic conditions^10^, although this may be possible under alkaline conditions. Interestingly, no systematic work has been undertaken to achieve lower oxidation state generation of soluble metal ions using an electrolytic cell without encountering problems with the water splitting reactions, although this feature is available in vanadium redox flow batteries which operate as a divided electrolytic cell in H_2_SO_4_ or CH_3_SO_3_H with a standard redox potential of 1.00 V (for V^5+^/V^4+^) and −0.26 V (for V^3+^/V^2+^)^11^. In practice, if electrolytic cells are to generate homogeneous mediators, they need to operate within the water splitting window and also at a potential far from the equilibrium value of their electrode reactions because of the inherent interfacial charge transfer and polarization resistances across the electrode-electrolyte interface^12^. In the case of the commercially available divided cell, this overpotential factor is substantial due to the properties of membrane separators like Nafion^®^^13^.

Water-electrolysis using a divided electrolytic cell creates a pH gradient in each half cell^14–16^. The following equations are relevant in acidic and alkaline media^17, 18^:

1 $${\rm{A}}{\rm{c}}{\rm{i}}{\rm{d}}{\rm{i}}{\rm{c}}:\quad 4{{\rm{H}}}^{+}+4{{\rm{e}}}^{-}\to 2{{\rm{H}}}_{2}({\rm{c}}{\rm{a}}{\rm{t}}{\rm{h}}{\rm{o}}{\rm{d}}{\rm{e}})$$

2 $$2{{\rm{H}}}_{2}{\rm{O}}\to 4{{\rm{H}}}^{+}+4{{\rm{e}}}^{-}+{{\rm{O}}}_{2}\,({\rm{anode}})\,({{E}}_{{\rm{e}}}^{0}=0.989\,{\rm{V}}\,({\rm{SCE}})$$

3 $${\rm{A}}{\rm{l}}{\rm{k}}{\rm{a}}{\rm{l}}{\rm{i}}{\rm{n}}{\rm{e}}:\quad 4{{\rm{H}}}_{2}{\rm{O}}+4{{\rm{e}}}^{-}\to 4{{\rm{O}}{\rm{H}}}^{-}+2{{\rm{H}}}_{2}({\rm{c}}{\rm{a}}{\rm{t}}{\rm{h}}{\rm{o}}{\rm{d}}{\rm{e}})({E}_{{\rm{e}}}^{0}=-1.063{\rm{V}}({\rm{S}}{\rm{C}}{\rm{E}})$$

4 $$4{{\rm{OH}}}^{-}\to 2{{\rm{H}}}_{2}{\rm{O}}+4{{\rm{e}}}^{-}+{{\rm{O}}}_{2}\,({\rm{anode}})$$

Because of the electrolyte pH change during water-electrolysis, the potential window tends to be widened by the −60 mV shift in E_H+/H2_ for every unit increase in pH^17^. The solution pH change also can alter some electrode potentials, and recently, a pH dependent water oxidation mechanism associated with a MnO_2_-modified electrode was reported^19^.

Morale-Guio *et al*. suggested that the use of either highly acidic or highly alkaline solutions can be used to favor the generation of an electrocatalyst in an electrochemical cell because of the potential window gain for the oxygen evolution reaction (OER) or hydrogen evolution reaction (HER)^20^. Hydrogen evolution is more facile when the concentration of protons is high, and oxygen evolution is easier when the hydroxide concentration is high^17^. Thus, in principle by combining the potential widening achieved by a pH change in both sides of an electrolytic cell, the simultaneous generation of both high and low oxidation state metal complexes (same or different metals) as two mediators for gas pollutant removal is facilitated.

The hypothesis that both high and low oxidation state mediators could be generated in one electrolytic experiment was tested in this study using the precursors, Co^II^SO_4_ (0.01 M) in 5 M H_2_SO_4_ in the anodic part of the cell and 0.01 M [Co^II^ (CN)_5_]^3−^ in 10 M KOH in the cathodic part to generate the Co^3+^ electrocatalyst in acid and Co^1+^ in base, respectively. The advantage of using [Co^II^(CN)_5_]^3−^ in the cathode cell is the ability to obtain a Co^1+^ electrocatalyst as a cyanide complex through a one electron reduction process in 10 M KOH medium without reduction to metallic cobalt; its p*K* value is approximately 18–19 ^21^. In initial electrolysis experiments the pH and half-cell potential variations were monitored during the electrolysis of water with 1 M NaCl as the electrolyte. Co^3+^ and Co^1+^ generation was tested using the precursor, Co^II^SO_4_, with acid in both half-cells and with acid in the anodic half-cell and base in the cathodic half-cell. The combination of Co^II^SO_4_ in acid as the anolyte and [Co^II^(CN)_5_]^3−^ in base and acid as the catholyte was also assessed for the simultaneous generation of Co^3+^ and Co^1+^. The electrodes selected for these experiments were a platinum coated titanium anode and a silver cathode. To assess the ability of electrochemically generated Co^3+^ and Co^1+^ to each remove one air-pollutant, the possibility of simultaneous electro-scrubbing of two gas pollutants CH_3_CHO and CCl_4_ was examined in a continuous mode of operation coupled with monitoring of the time dependence of the concentrations of pollutants with an online Fourier transform infrared (FTIR) gas analyzer. The results highlight the importance of pH and broadening of the potential window to generate two mediators simultaneously.

## Results and Discussion

### Probing pH and potential changes during water-electrolysis

**Figure 1:**
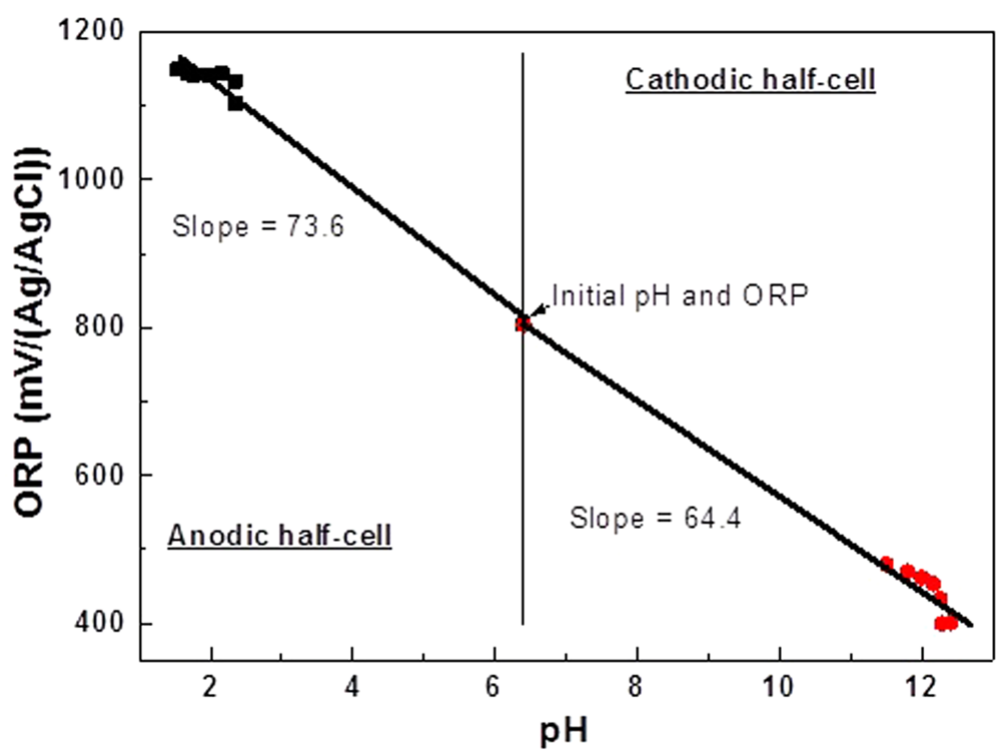
Half-cell potential dependence on pH. pH dependent half-cell variation during water electrolysis in the presence of 1 M NaCl. Conditions: Electrolyte volume is 200 ml (both sides); Electrodes are Pt coated Ti (anode, 4 cm^2^) and Ag (cathode, 4 cm^2^); current density = 50 mA cm^−2^; electrolyte flow rate = 70 ml min^−1^; membrane is Nafion 324.

First of all, 1 M NaCl was added as the electrolyte to both half cells and the changes in pH and half-cell potential that occurred during electrolysis of water were determined. Figure 1 shows that the initial oxygen reduction potential (ORP) value of 804 mV changed within 3 min to 480 mV and 1104 mV in the cathodic and anodic half-cells, respectively, and then slowly approached a steady state value. At the same time, the initial pH value of 6.4 changed rapidly to 11.5 and 2.35 within 3 min in the cathodic and anodic half-cells respectively and became constant after 5 h of electrolysis. The anodic half-cell slope value of 73.6 mV/pH and the cathodic half-cell slope value of 64.4 mV/pH based on assumption of a linear relationship during the initially rapidly changing initial 3 min showed a good match to the Pourbaix plot for water electrolysis^22^. The changes in pH and half-cell potential during water-electrolysis in the divided electrolytic cell confirms that in a practical electrolytic cell with dual mediators it is advantageous to generate one of them in acid conditions in the anodic half-cell and the other under alkaline conditions in the cathodic half-cell.

### Acid pH in both half-cells generates only the Co^3+^ mediator

**Figure 2:**
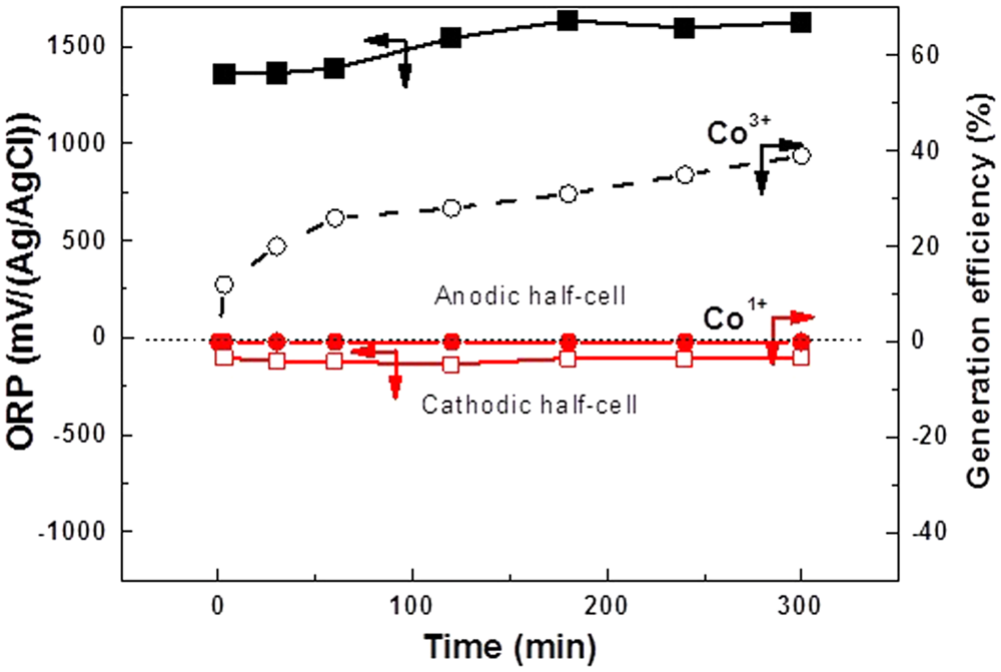
Co^3+^ and Co^1+^ generation with acid in both half-cells. ORP (■, □) and generation efficiency (○, ●) found during the course electrolysis of Co^II^SO_4_ with 5 M H_2_SO_4_ in both cells. Conditions: Initial Co^II^SO_4_ = 0.01 M; Other experimental conditions are as in Fig. 1.

When Co^II^SO_4_ was placed in 5 M H_2_SO_4_ in both half cells, as applies in Fig. 2, the ORP changed from 929 mV to 1624 in the anodic half-cell. Oxidative formation of Co^3+^ (reaction 5) was confirmed by conventional potentiometric titrations, and an oxidation efficiency of 39% was achieved. However, there was no change in the ORP, (−110 ± 10 mV reached in 1 min), in the cathodic half-cell during the 5 h electrolysis time implying no cathodic reduction occurred to give Co^1+^ (potentiometric titrations were not possible), and that dominant H_2_ evolution occurs, as shown in reaction 6.

5 $${\rm{At}}\,\,{\rm{anode}}:\quad {{\rm{Co}}}^{2+}\to {{\rm{Co}}}^{3+}+{{\rm{e}}}^{-}\,({{E}^{0}}_{{\rm{Co}}3+/{\rm{Co}}2+}=1.602\,{\rm{V}}\,({\rm{SCE}}))$$

6 $${\rm{At}}\,\,{\rm{cathode}}:\quad 2{{\rm{H}}}^{+}+2{{\rm{e}}}^{-}\to {{\rm{H}}}_{2}({\rm{g}})\,({{E}^{0}}_{{\rm{H}}+/{\rm{H}}2}=-\,0.240({\rm{SCE}}))$$

### Acid and base conditions respectively in the anodic and cathodic half-cells generate both the Co^3+^ and Co^1+^ mediators

**Figure 3:**
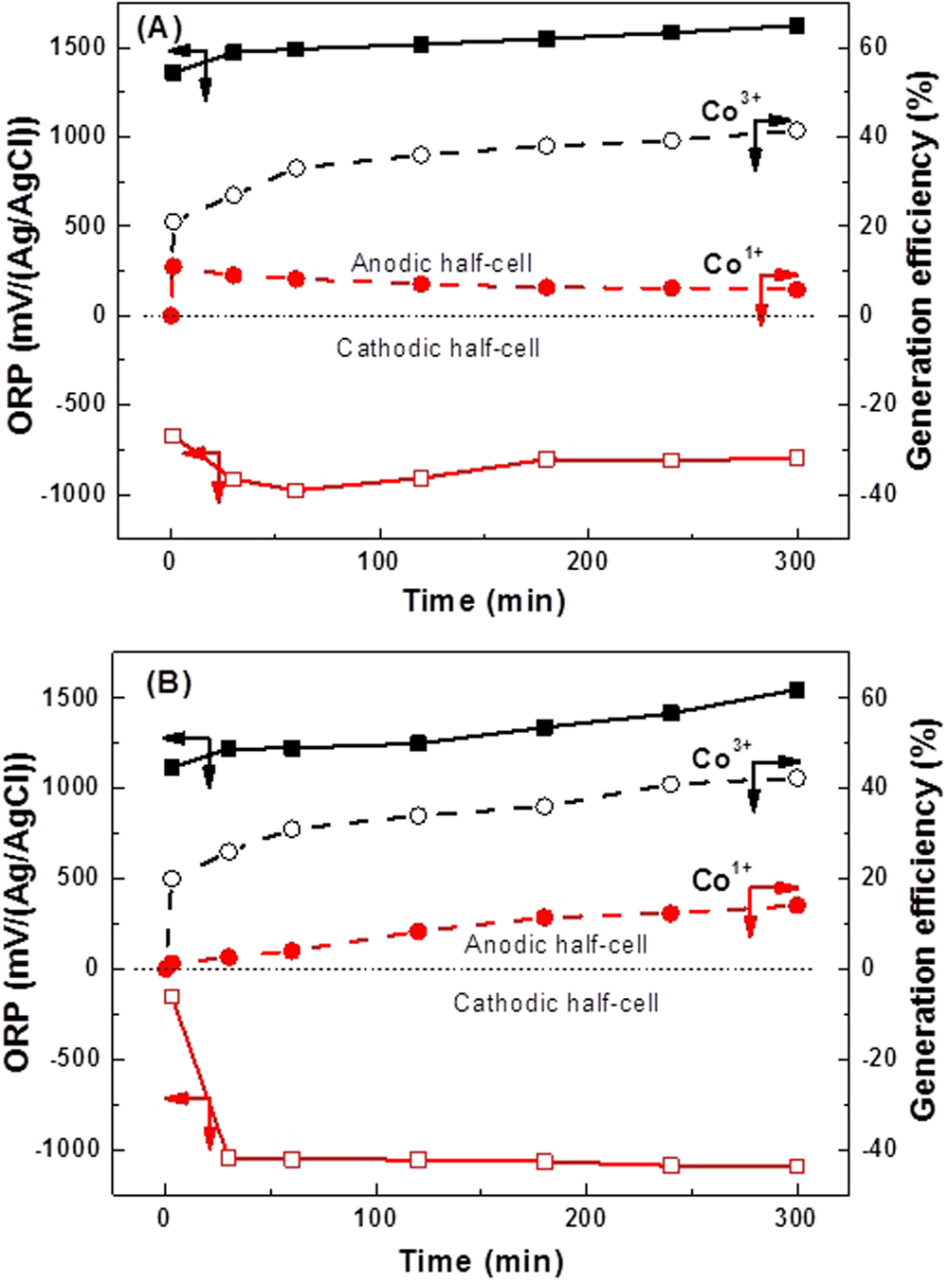
Co^3+^ and Co^1+^ generation with acid and base in the half-cells on. ORP (■, □) and generation efficiency (○, ●) found in each half-cell during electrolysis with (**A**) 0.01 M Co^II^SO_4_ in both half-cells or (**B**) 0.01 M Co^II^SO_4_ in the anodic half-cell and 0.01 M [Co^II^(CN)_5_]^3−^ in the cathodic half-cell. Conditions: Anolyte = 5 M H_2_SO_4_; Catholyte = 10 M KOH; Other experimental conditions are as in Fig. 1.

As inferred from analysis of the above water-electrolysis experimental data, for dual mediator generation, 0.01 M Co^II^SO_4_ should be placed in 5 M H_2_SO_4_ in the anodic half-cell and in 10 M KOH in the cathodic half cell. The electrolysis results (Fig. 3A) now exhibit a gradual increase in the ORP up to a maximum value 1624 mV in anodic part, with 1800 mV being standard potential under ideal conditions^5^) and decrease to a minimum value of −978 mV in cathodic part, with −1200 mV being the standard potential^21^), consistent with the formation of Co^3+^ and Co^1+^ at the relevant half-cells during electrolysis. Although the generation efficiency of Co^3+^ was 41% in 5 h, the generation efficiency of Co^1+^ initially reached only 11% then decreased to 8%. In highly basic media (>6 M) Co^2+^ becomes [Co^II^(OH)_4_]^2−^ ^23^, which may be reduced electrochemically to low-valent [Co^I^(OH)_4_]^3−^.

Figure 3B shows the electrolysis results when Co^II^SO_4_ and [Co^II^(CN)_5_]^2−^ are present in the anodic and cathodic half cells containing 5 M H_2_SO_4_ and 10 M KOH as the electrolytes respectively. The initial shift in the ORP of the electrolyzed solution at each half-cell to 1.35 V and −1.05 V in 30 min highlights the formation of higher oxidation state Co^3+^ and lower oxidation state Co^1+^ in the relevant half-cells. This result confirms that the pH change and potential widening effect can allow the generation of two homogenous mediators concurrently using a single electrolytic cell. A [Co^II^(CN)_5_]^3−^ to [Co^I^(CN)_5_]^4−^ reduction process occurs under highly alkaline conditions at Hg electrode as shown in equations 7 and 8 with *E* ^0^ = −1.2 V (SCE) applying to 4 M and higher KOH concentrations^21, 24^. A similar electrochemical reduction process is expected to occur at a Ag metal electrode with high KOH concentrations,

7 $${\rm{A}}{\rm{t}}{\rm{t}}{\rm{h}}{\rm{e}}{\rm{c}}{\rm{a}}{\rm{t}}{\rm{h}}{\rm{o}}{\rm{d}}{\rm{e}}:{[{{\rm{C}}{\rm{o}}}^{{\rm{I}}{\rm{I}}}{({\rm{C}}{\rm{N}})}_{5}]}^{3-}+{{\rm{e}}}^{-}\to {[{{\rm{C}}{\rm{o}}}^{{\rm{I}}}{({\rm{C}}{\rm{N}})}_{5}{\rm{H}}]}^{3-}({\rm{i}}{\rm{n}}{\rm{a}}{\rm{c}}{\rm{t}}{\rm{i}}{\rm{v}}{\rm{e}}{\rm{w}}{\rm{i}}{\rm{t}}{\rm{h}}[{\rm{K}}{\rm{O}}{\rm{H}}]{\rm{l}}{\rm{e}}{\rm{s}}{\rm{s}}{\rm{t}}{\rm{h}}{\rm{a}}{\rm{n}}4{\rm{M}})$$

8 $${[{{\rm{C}}{\rm{o}}}^{1+}{({\rm{C}}{\rm{N}})}_{5}{\rm{H}}]}^{3-}+{{\rm{O}}{\rm{H}}}^{-}\to {[{{\rm{C}}{\rm{o}}}^{1+}{({\rm{C}}{\rm{N}})}_{5}]}^{4-}+{{\rm{H}}}_{2}{\rm{O}}(-1.2{\rm{V}}({\rm{S}}{\rm{C}}{\rm{E}}){\rm{w}}{\rm{i}}{\rm{t}}{\rm{h}}[{\rm{K}}{\rm{O}}{\rm{H}}]{\rm{g}}{\rm{r}}{\rm{e}}{\rm{a}}{\rm{t}}{\rm{e}}{\rm{r}}{\rm{t}}{\rm{h}}{\rm{a}}{\rm{n}}4{\rm{M}})$$

In additional experiments it was shown that with [Co^II^(CN)_5_]^3−^ in 5 M H_2_SO_4_, no change in the cathodic ORP occurred, as expected if no low-valent Co^1+^ formation had occurred (Fig. SI 1). This result confirms that high pH is essential in the cathodic half-cell in order to generate low-valent Co^1+^ mediators.

To confirm that [Co^II^(CN)_5_]^3−^ reduction can occur in the electrolysis conditions used above, experiments using cyclic voltammetry (CV) was undertaken in both acidic and basic media at a macrodisk Ag electrode (Fig. SI 2). The CV method showed that reduction of [Co^II^(CN)_5_]^3−^ does occur (a reduction peak at −830 mV) in alkaline media (Fig. SI 2 **curve b**) whereas H_2_ evolution is favoured under acid conditions (Fig. SI 2 risinng part of curve a near 0 V) highlighting the need for a high pH in the cathodic half-cell in order to generate 2 mediators simultaneously. Oxidation of [Co^II^(CN)_5_]^3−^ (not relevant to this paper) at about 0.65 V also is evident in curve b of Fig. SI 2. Importantly, the efficiency dependence on time (Fig. SI 3) **data** shows that when Co^1+^ and Co^3+^ are both generated simultaneously at the cathode and anode half cells, respectively, that the Co^1+^ yield increased when the current density increased from 20 to 30 mA cm^−2^ then decreased when a higher current density of 50 mA cm^−2^ was employed due to enhanced formation of inactive [Co(CN)_5_H]^3−^ (eqn. 7) or further reduction to cobalt metal. In contrast, the Co^3+^ yield increased progressively with increasing current density as in our previous report^25^. The concentrations of Co^1+^ and Co^3+^ were 2.1 mM and 3.50 mM, respectively, after 6 h electrolysis at a current density of 50 mA cm^−2^. This shows that Co^3+^ and Co^1+^ formation competes effectively with gas evolution at both the anode and cathode during electrolysis.

The efficiency of the generation of two mediators concurrently is an important practical consideration in an electrolytic cell. As shown in Fig. 3, the generation efficiency (current efficiency for the long term electrolysis (5 h) in dichloroethane is only 6%^26^) of Co^3+^ reached 41% at the anodic half-cell after 5 h operation. With the help of the cathodic half-cell operation, an additional 15% generation efficiency of Co^1+^ formation was achieved to give a total of 56%, with this new approach.

### Probing the membrane stability under highly acidic and basic conditions

Although Nafion 324 is used in the chloralkali process^27, 28^, the stability of the membrane was tested under the present harsh conditions. No prominent changes were observed in SEM images on either side of the membrane surface after electrolysis (Fig. SI 4) although evidence for the deposition of salts containing potassium, sulfur, aluminum, chlorine, and cobalt was obtained that in some cases might have come from impurities present in the supporting electrolyte. In further evaluation studies, four consecutive batch electrolysis runs were performed with Co^3+^ and Co^1+^ generation, to give the data provided in Fig. SI 5. The Co^3+^ and Co^1+^ concentrations observed remain constant within experimental error, which confirms the stability of the membrane under the harsh conditions employed.

### Application to air-pollutants

Using the simultaneous generation of the Co^3+^ and Co^1+^ homogeneous mediators, two air-pollutants were removed by MEO and mediated electrocatalytic reduction (MER) using electro-scrubbing. Although the two mediators can be produced concurrently, quantitative assessment of air-pollutant removal by electro-scrubbing had to be undertaken by a stepwise approach due to practical limitations with the availability of only one online FTIR gas analyzer.

**Figure 4:**
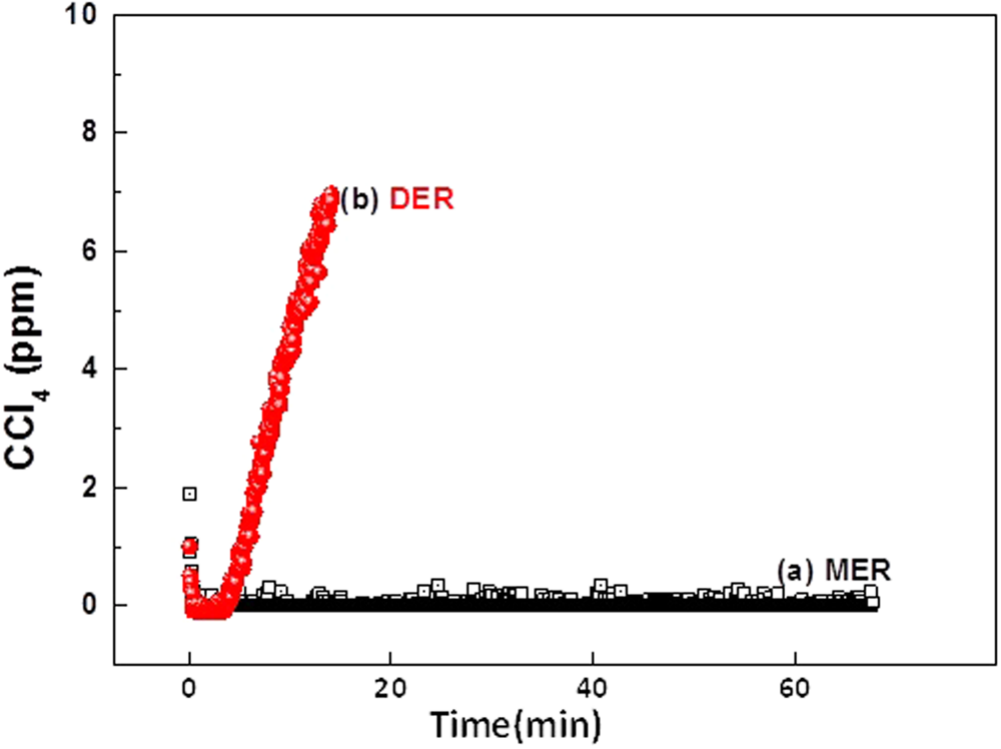
Cathodic half-cell application for CCl_4_ gas pollutant removal. Effect of electrogenerated Co^3+^ in the cathodic half-cell on removal of gaseous CCl_4_ as a function of electrolysis time with different electro-scrubbing processes: (a) MER using [Co^I^(CN)_5_]^4−^ in 10 M KOH; (b) DER using 10 M KOH. Electro-scrubbing conditions: Gas flow rate = 0.2 L min^−1^; Liquid low rate = 3 L min^−1^; Feed CCl_4_ = 8 ppm. Electrolysis conditions: Electrodes = Pt coated Ti (anode) and Ag (cathode) (50 cm^2^ area); Electrolyte volume = 500 ml (each side); solution flow rate = 2 L min^−1^; Current density = 30 mA cm^−2^.

The electrolytically-formed Co^1+^ in the cathodic compartment under the optimized current density was used to degrade CCl_4_ in a continuous process. Note that Chloro Volatile Organic Compunds (CVOCs) like CCl_4_ cannot be degraded at the anodic half-cell due to electrode fouling by polymerization^29^. When the concentration of Co^1+^ reached 14%, ≈8 ppm of CCl_4_ was injected at a gas flow rate of 0.2 L min^−1^ through the bottom of the scrubber at a liquid flow rate of 3 L min^−1^ with a closed loop catholyte circulation. As shown in Fig. 4 **curve a**, a rapid decrease in the outlet CCl_4_ to 0.3 ppm was observed and this trend was maintained for up to 1 h of operation, which is equal to 96% removal or 0.53 mg h^−1^ of CCl_4_ removed and 12.78 mg day^−1^ can be achieved if the same removal rate continues. In the absence of Co^1+^ in the electrolyte, the direct electrocatalytic reduction (DER) of CCl_4_ does not occur in the electrolyzed 10 M KOH solution. In this case (Fig. 4 **curve b**) an initial decrease is detected in the outlet followed by an increase with time, and ultimately returning to the level of the inlet (≈8 ppm), which equates to 0% removal. These data confirm that the CCl_4_ removal process follows an MER pathway, as shown in reaction 9.

9 $$({\rm{with}}\,10\,{\rm{M}}\,{\rm{KOH}})\,{[{{\rm{Co}}}^{{\rm{I}}}{({\rm{CN}})}_{5}]}^{4-}+{{\rm{CCl}}}_{4}\to {[{{\rm{Co}}}^{{\rm{II}}}{({\rm{CN}})}_{5}]}^{3-}+{\rm{product}}({\rm{s}})$$

Additionally, the byproducts identified to accompany CCl_4_ removal by electro-scrubbing for I hour are chloride (Cl^−^) in solution and CO_2_ in the gas phase. In brief, the chloride concentration in the catholyte solution phase increases from about 1 to 1.66 g L^−1^ as shown in Fig. S 16. At the same time, the CO_2_ concentration in the gas phase increases to around the 30 to 40 ppm level and all CCl_4_ is removed as shown by increase in the gas phases FTIR CO_2_ stretching response in the 2289.9 to 2385.5 cm^−1^ range and a decrease in the CCl_4_ FTIR stretching response in the region 737.12–815.74 cm^−1^ (reference made to standard FTIR gas phase spectra available in the MIDAC library).

**Figure 5:**
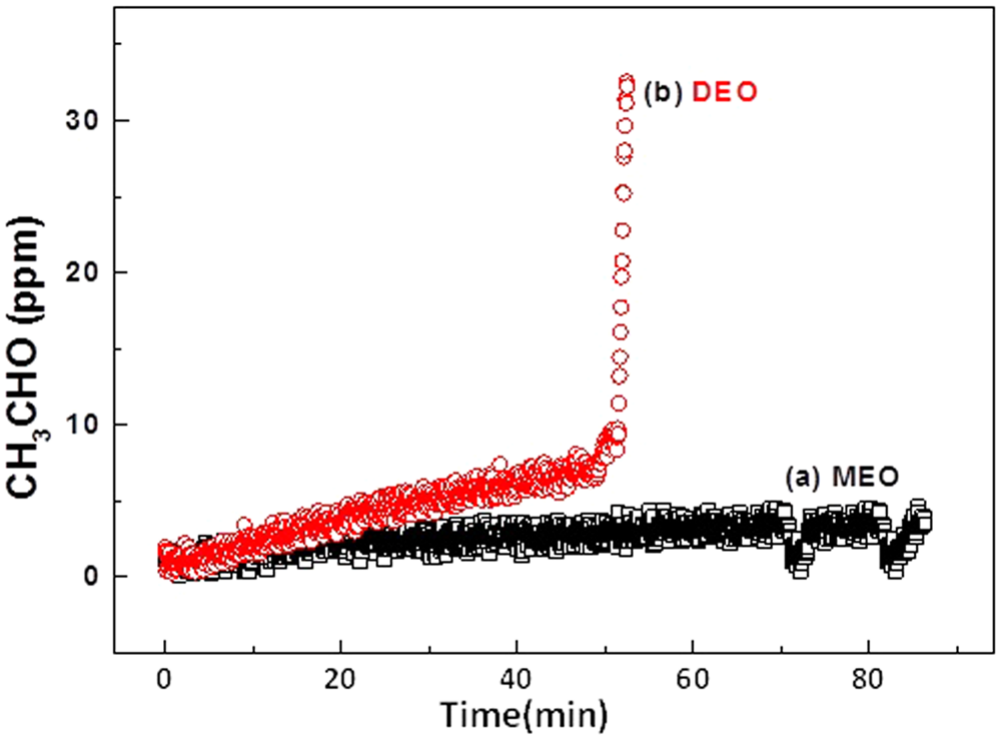
Anodic half-cell application for CH_3_CHO gas pollutant removal. Effect of electrogenerated Co^3+^ in the anodic half-cell on the removal of gaseous CH_3_CHO as a function of electrolysis time with different electro-scrubbing processes: (a) MEO using Co^3+^ in 5 M H_2_SO_4_; (b) DEO using 5 M H_2_SO_4_. Electro-scrubbing conditions: Gas flow rate = 0.2 L min^−1^; Liquid low rate = 3 L min^−1^; Feed CH_3_CHO = 30 ppm. Electrolysis conditions are the same as in Fig. 4.

In an anodic half-cell, electrolytically generated Co^3+^ was used to degrade CH_3_CHO through a continuous process. When the Co^3+^ concentration reached 41% by electrolytic generation, ≈30 ppm of CH_3_CHO was injected at a gas flow rate of 0.2 L min^−1^ through the bottom of the scrubber at a liquid flow rate of 3 L min^−1^ with closed loop circulation. As shown in Fig. 5 **curve a**, a rapid fall in the outlet CH_3_CHO to a concentration of 3 ppm (90% removal) occurs with this pattern continuing for up to 1 h of operation. The possibility of direct electrocatalytic oxidation (DEO) of CH_3_CHO (without presence of Co^3+^) was considered in an electrolyzed solution of 5 M H_2_SO_4_ (Fig. 5 **curve b**). In this case, the initial decrease in the outlet concentration of CH_3_CHO ceased after 35 min, due to electrode fouling during DEO of CH_3_CHO^25^. In contrast, continuous removal of CH_3_CHO was achieved in the presence of electrogenerated Co^3+^ which confirms the removal process follows the MEO pathway without any additional reactions as shown in equation 10.

10 $${{\rm{Co}}}^{3+}+{{\rm{CH}}}_{3}{\rm{CHO}}\to {{\rm{Co}}}^{2+}+{\rm{product}}({\rm{s}})$$

## Disscussion

In this investigation, widening of the potential range available by controlling the conditions used for water electrolysis has been utilised to generate two mediators simultaneously that facilitate effective air-pollutant removal, with one formed in each half cell. In a practical demonstration of the dual mediator generation approach, the degradation of one pollutant (CCl_4_) at the cathode and one (CH_3_CHO) at the anode was achieved simultaneously with efficiencies in both cases being greater than 90%.

## Methods

### Preparation of the cobalt cyanide complex

The cobalt cyanide complex ([Co^II^(CN)_5_]^3−^) was synthesized according to a literature procedure^30^. Briefly, 53.8 g of potassium cyanide dissolved in 50 ml water was added to 60 ml of a cooled solution (4 °C) containing 40.0 g of Co^II^(NO_3_)_2_ under a nitrogen atmosphere (∼6.2 cyanide anions per cobalt), after which an equal volume of chilled alcohol was added. The resulting mixture was chilled slowly until thin violet platelets of the cobalt cyanide complex appeared. The solid was collected by rapid filtration, washed with cold alcohol, dried in a vacuum desiccator, and then stored in an air-tight brown bottle.

### Electrolytic cell

The electrolytic flow cell used for the simultaneous generation of Co^1+^ at the cathode and Co^3+^ at the anode was a plate-and-frame type, narrow gap, divided configuration^4^. The mesh type Pt-coated-Ti anode and a Pt/Ag, cathode were separated by a Nafion® 324 membrane with the inter-electrode gap being maintained at 5 mm by two Viton rubber gaskets (2 mm thickness). The electrode assembly was clamped tightly with Ti end plates using a filter press technique. Separate channel paths were available in each compartment through which the anolyte and catholyte solutions could flow across their relevant electrodes. A 0.2 L solution of Co^II^SO_4_ (10 mM) in 5 M H_2_SO_4_ and 0.2 L of each of Co^II^SO_4_ in 5 M H_2_SO_4_, Co^II^SO_4_ in 10 NaOH, [Co^II^(CN)_5_]^3−^ (10 mM) in 5 M H_2_SO_4_ or 10 M KOH were placed in separate anolyte and catholyte tanks, respectively. The anolyte and catholyte solutions were circulated continuously through the anode and cathode compartments of the electrochemical cell at a constant flow rate of 70 ml min^−1^ using peristaltic pumps (Masterflex - L/S, Model No. 7524-45, Cole Parmer Instrument Company, USA). The active Co^1+^ and Co^3+^ mediators were generated galvanostatically by applying constant current densities between 10 to 50 mA cm^−2^ using a DC power supply **(**Korea Switching Instruments). The effective surface area of each electrode exposed to the solution was 4 cm^2^. All measurements were carried out at 20 ± 2 °C.

During gas-pollutant removal, a scrubber column (40 cm high and 5.5 cm (i.d.)) packed with 1 cm^2^ of Teflon tubes was attached to top of the anolyte and catholyte tanks respectively, which were already attached to the flow through electrolytic divided cell, as shown in Fig. SI 7. The cell had cathode and anode areas of 50 cm^2^ and an electrolyte volume of 500 ml. The scrubbing system consisted of an air supply system, a scrubbing solution ([Co^II^(CN)_5_]^3−^/[Co^I^(CN)_5_]^4−^ in a KOH solution or Co^2+^/Co^3+^ in H_2_SO_4_ solution), a scrubbing reactor column, and an FTIR gas analyzer system (MIDAC Corporation, USA) equipped with a data logger. The anolyte and catholyte solutions were circulated continuously at a flow rate of 2 L min^−1^ using magnetic pumps (Pan World Co., Taiwan). For electro-scrubbing, the activated solutions were pumped separately into the scrubber column at a flow rate of 3 L min^−1^. CH_3_CHO and CCl_4_ gases (from RIGAS (1000 ppm), Korea) and air mixtures, which were obtained by the controlled mixing of air and CH_3_CHO or CCl_4_ gas using mass flow controllers (MFCs), were introduced to the relevant bottom of the scrubbers at a set gas flow rate. The CH_3_CHO or CCl_4_ gas to air ratios obtained using the MFCs were confirmed prior to undertaking the experiments. The scrubbing solution was re-circulated through the electrochemical cell to regenerate Co^1+^ or Co^3+^. Before starting the CH_3_CHO or CCl_4_ removal experiment, the electrochemical cell was operated until Co^1+^ or Co^3+^ conversions reached nearly a steady state, and the scrubbing solution was then pumped into the scrubber column.

Cyclic voltammetry was carried out with a PARC VersaSTAT 3 instrument in a glass cell furnished with two compartments separated by a membrane. A platinum mesh and a dip type Ag/AgCl electrode were used as the counter and reference electrodes, respectively. Ag was used as working electrode, and were placed along with reference electrode in one of the cell compartments (acid or base as appropriate), while the counter electrode was placed in the other compartment.

### Estimation of Co^1+^ and Co^3+^ concentrations

Aliquots (5 ml) of the catholyte containing Co^1+^ were drawn periodically during the course of electrolysis experiments and the generated Co^1+^ concentration was calculated by titration against a standard [Fe^III^(CN)_6_]^3−^ (1 mM) solution. The redox potential of the Co^1+^-containing solution was measured using an oxidation reduction potential (ORP) electrode (Model No. EMC 133, iSTEK, USA (6 mm Pt with Ag/AgCl reference electrode and gel electrolyte) with data obtained with a pH/ISE meter (iSTEK, pH-240L, USA) used to identify the end point during the titration. During each titration, [Fe^III^(CN)_6_]^3−^ addition was stopped when the measured ORP value, which shifted to negative values with increasing Co^1+^ concentration, reverted to its initial value measured before electrolysis. The concentration of Co^1+^ was calculated from that of [Fe^III^(CN)_6_]^3−^ consumed. In a similar manner, the Co^3+^ concentration from the anode compartment was obtained by titration with Fe^II^SO_4_ (1 mM). Here, the initial ORP value of the Co^3+^-containing solution, which was approximately 600 mV, became increasingly positive with increasing Co^3+^ concentration. The concentration of Co^3+^ was calculated from that of the Fe^II^SO_4_ titrant. The concentrations determined in this manner were reproducible to ±0.02 mM.

#### Chloride ion analysis

The procedure used is based on that described in the user’s manual provided by HACH with their Model DR-2800 spectrophotomer. 5 ml of sample drawn from the electrolysis solution (catholyte) was neutralized with perchloric acid by titration and the resultant solution centrifuged to remove any precipitate present. If required, a final adjustment of the pH to 6–7 was made using 1 M NaOH. 0.8 ml of mercury thiocyanate (HACH reagent) and 0.6 ml of 1 M ferric perchlorate were then added to give a brownish yellow solution. The chloride content of this solution was then measured colorimetrically and the finally reported value determined after correction for dilution.

## Electronic supplementary material

Supplementary Information
