# Supplementary material for: Implementation of concurrent electrolytic generation of two homogeneous mediators under widened potential conditions to facilitate removal of air-pollutants
Source: Sci Rep. 2017 Feb 13;7:29. doi: 10.1038/s41598-017-00058-2 (PMC5428318; doi:10.1038/s41598-017-00058-2)
Supplement: Supplementary file 1 — Supplementary Information [file 41598_2017_58_MOESM1_ESM.pdf]

# Implementation of concurrent electrolytic generation of two homogeneous mediators under widened potential conditions to facilitate removal of air-pollutants

**Govindan Muthuraman,[1] Alan M. Bond,[2] and Moon Il-Shik[1*]**
[1] Department of Chemical Engineering, Sunchon National University, 255-Jungang ro, Suncheon-si, Jeollanam-do, 57922, South Korea.
[2] School of Chemistry, Monash University, Clayton, Victoria 3800, Australia

**Fig.SI 1**

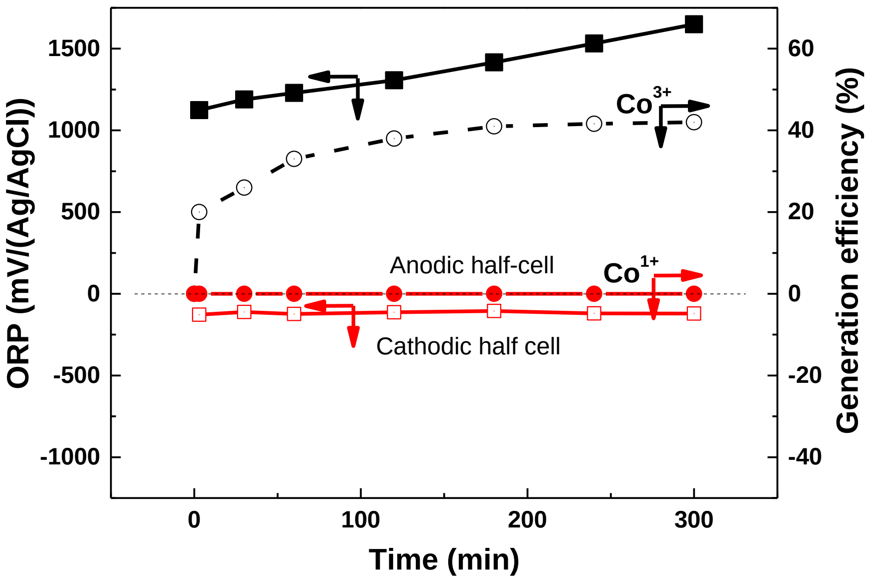


**Fig.SI 1 $Co^{3+}$ and $Co^{1+}$ generation with 5 M $H_2SO_4$ present in both half-cells.** ORP (■,□) and generation efficiency (○,●) during the electrolysis of 0.01 M $Co^{II}SO_4$ in the anodic half-cell and 0.01 M $[Co^{II}(CN)_5]^{3-}$ in the cathodic half-cell. Conditions: Electrolyte volume = 200 ml (both sides); Electrode = Pt coated Ti (anode, 4 $cm^2$) and Ag (cathode, 4 $cm^2$); Current density = 50 mA $cm^{-2}$; Electrolyte flow rate = 70 ml $min^{-1}$; Membrane = Nafion 324.

**Fig.SI 2**

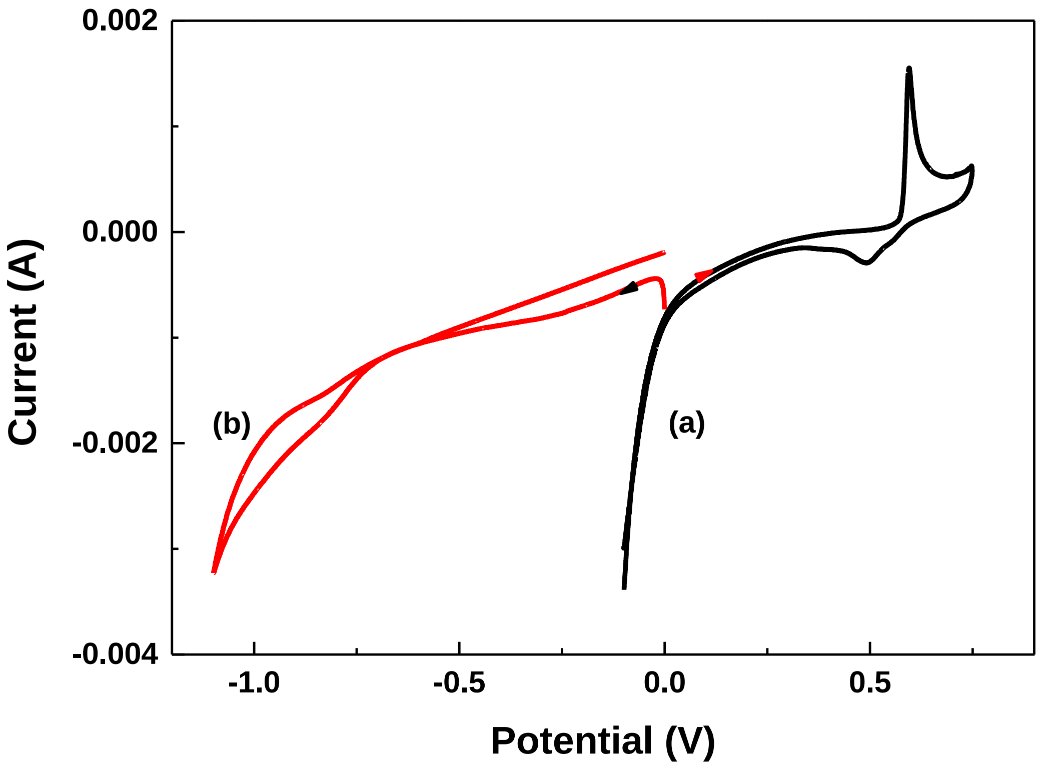


**Fig.SI 2 Cyclic voltammetry of 0.01 M** $[Co^{II}(CN)_5]^3$ **under acidic and basic electrolyte conditions**. (a) 3 M $H_2SO_4$ and (b) 10 M KOH. (Conditions: scan rate = 20 mV $s^{-1}$; Working electrode = Ag.

Fig.SI 3

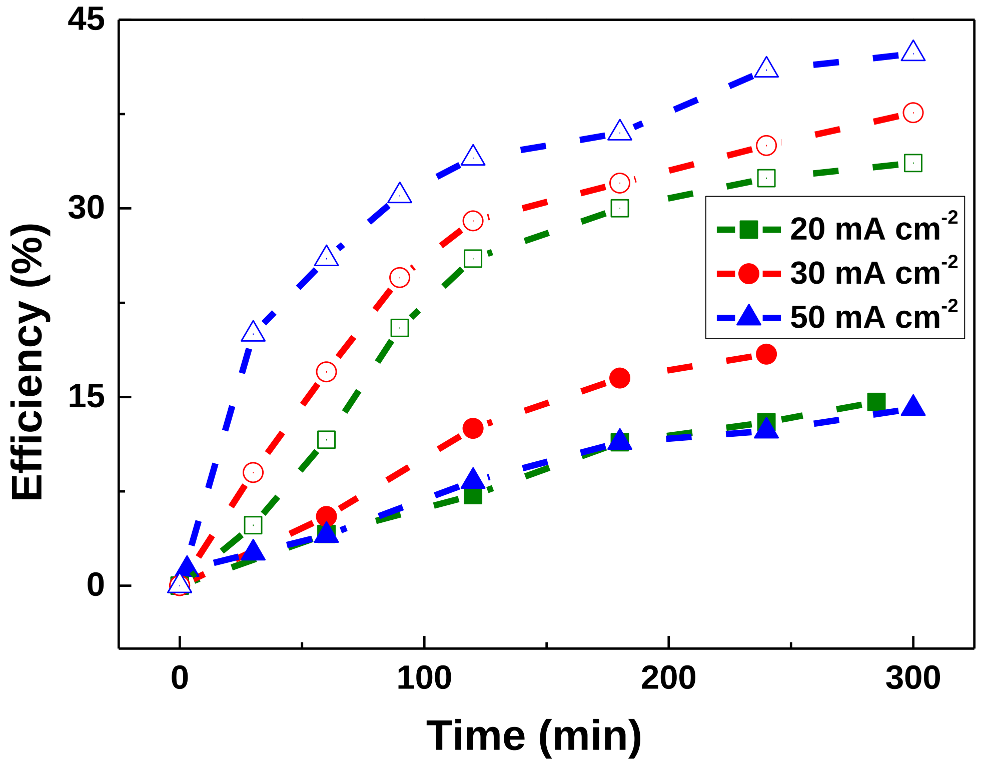


**Fig.SI 3 Dependence of current density on 0.01 M $[Co^{II}(CN)_5]^{3-}$ reduction (generation of $Co^{1+}$, closed symbol) and 0.01 M $Co^{II}SO_4$ oxidation (generation of $Co^{3+}$, open symbol) efficiencies$^-$ in 10 M KOH and 5 M $H_2SO_4$, respectively, as a function of current density**. Conditions: Electrolyte volume = 200 ml (both sides); Electrode = Pt coated Ti (anode, 4 $cm^2$) and Ag (cathode, 4 $cm^2$); Electrolyte flow rate = 70 ml $min^{-1}$; Membrane = Nafion 324.

**Fig.SI 4**

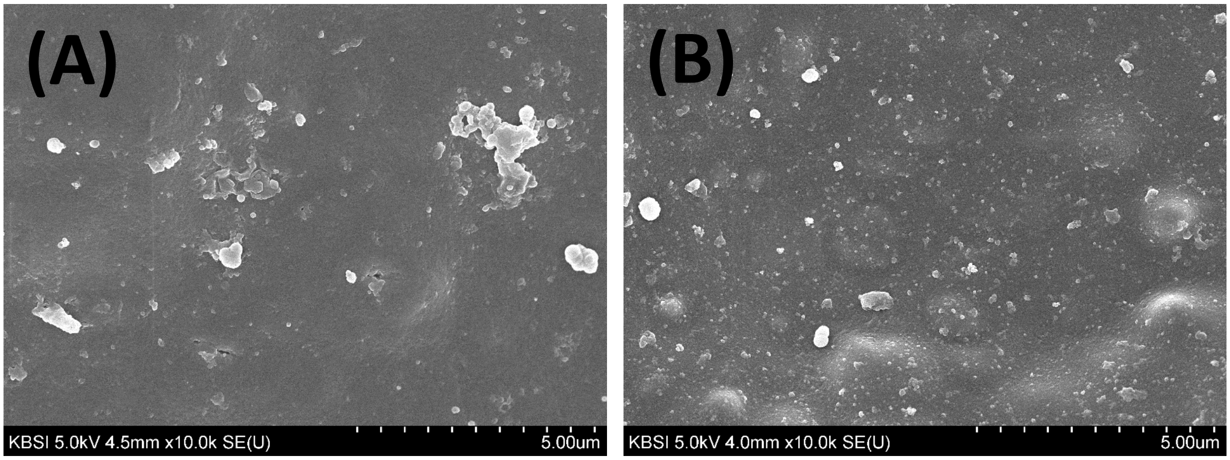


**Fig.SI 4** SEM images of the sides of the Nafion 324 membrane facing the anodic (A) and cathodic (B) half-cell solutions after electrolysis. Electrolysis conditions: Electrolyte volume = 200 ml (both sides); Electrolyte = 0.01 M $Co^{II}SO_4$ in 5 M $H_2SO_4$ (anodic half-cell) and 0.01 M $[Co^{II}(CN)_5]^{3-}$ in 10 M KOH (cathodic half-cell); Electrode = Pt coated Ti (anode, 4 $cm^2$) and Ag (cathode, 4 $cm^2$); Current density = 50 mA $cm^{-2}$; Electrolyte flow rate = 70 ml $min^{-1}$.

**Fig.SI 5**

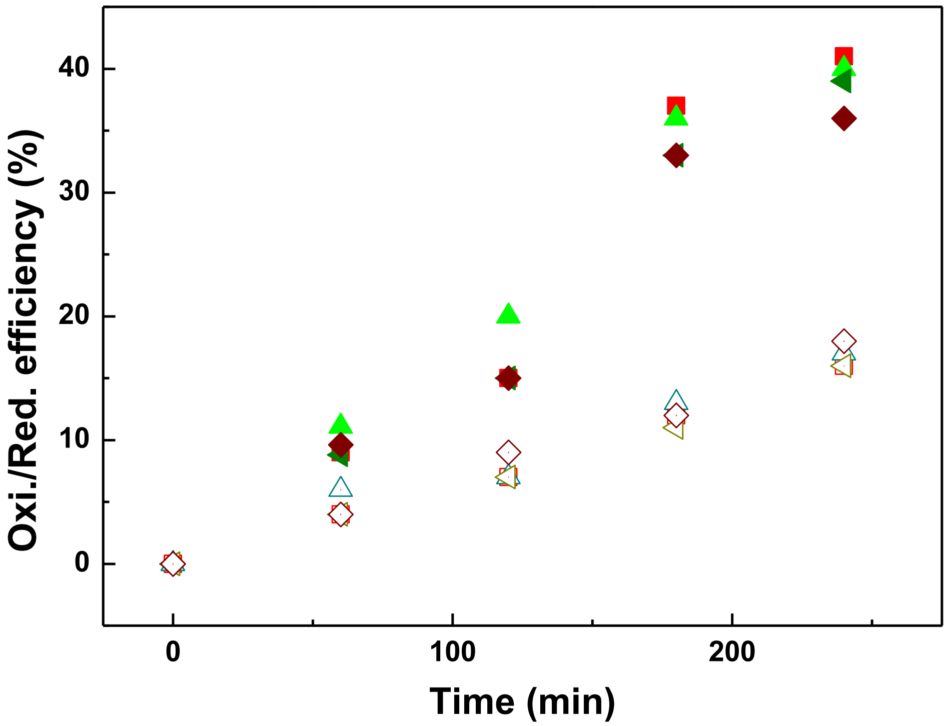


**Fig.SI 5 Nafion 324 membrane durability testing.** Oxidation (generation of $Co^{3+}$, closed symbols) and reduction (generation of $Co^{1+}$, open symbols) efficiency data obtained from four consecutive electrolysis batch experiments. Conditions: Electrolyte volume = 200 ml (both sides); Electrolyte = 5 M $H_2SO_4$ (anodic half-cell) and 10 M KOH (cathodic half-cell); Electrode = Pt coated Ti (anode, 4 $cm^2$) and Ag (cathode, 4 $cm^2$); Current density = 50 mA $cm^{-2}$; Electrolyte flow rate = 70 ml $min^{-1}$; Membrane = Nafion 324.

**Fig.SI 6**

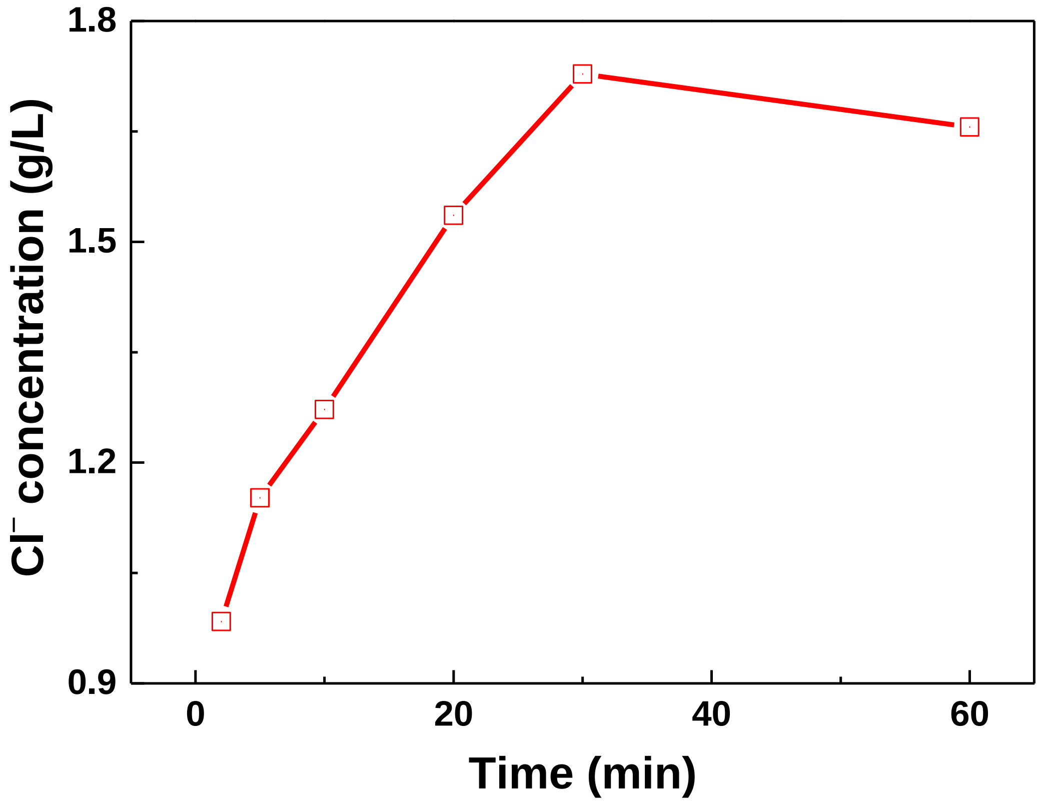


**Fig.SI 6 Chloride ion concentration variation in catholyte solution during removal of gas $CCl_4$ using electrogenerated $Co^{1+}$ from 0.01 M $[Co^{II}(CN)_5]^{3-}$ at electro-scrubbing.** Conditions: Electrolyte volume = 200 ml (both sides); Electrolyte = 5 M $H_2SO_4$ (anodic half-cell) and 10 M KOH (cathodic half-cell); Electrode = Pt coated Ti (anode, 4 $cm^2$) and Ag (cathode, 4 $cm^2$); Electrolyte flow rate = 70 ml $min^{-1}$; Membrane = Nafion 324.

**Fig.SI 7**

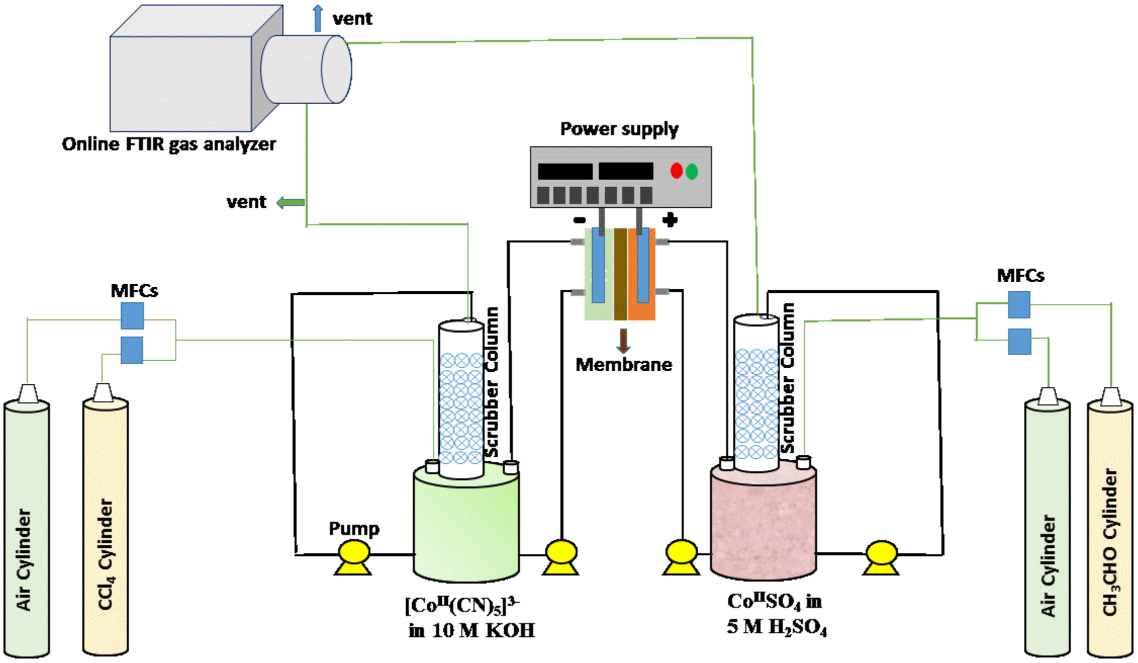


**Fig. SI 7** **MEO/MER operation**. A schematic diagram illustrating the simultaneous removal of air pollutants by the MEO/MER process using electro-scrubbing.
